# Advanced Neuromonitoring and Imaging in Pediatric Traumatic Brain Injury

**DOI:** 10.1155/2012/361310

**Published:** 2012-05-21

**Authors:** Stuart H. Friess, Todd J. Kilbaugh, Jimmy W. Huh

**Affiliations:** ^1^Department of Anesthesiology and Critical Care, Children's Hospital of Philadelphia, University of Pennsylvania Perelman School of Medicine, Critical Care Office 7C10, 7 South Tower, Philadelphia, PA 19104, USA; ^2^Department of Anesthesiology and Critical Care, Children's Hospital of Philadelphia, University of Pennsylvania Perelman School of Medicine, Critical Care Office 7C08, 7 South Tower, Philadelphia, PA 19104, USA; ^3^Department of Anesthesiology and Critical Care, Children's Hospital of Philadelphia, University of Pennsylvania Perelman School of Medicine, Critical Care Office 7C06, 7 South Tower, Philadelphia, PA 19104, USA

## Abstract

While the cornerstone of monitoring following severe pediatric traumatic brain injury is serial neurologic examinations, vital signs, and intracranial pressure monitoring, additional techniques may provide useful insight into early detection of evolving brain injury. This paper provides an overview of recent advances in neuromonitoring, neuroimaging, and biomarker analysis of pediatric patients following traumatic brain injury.

## 1. Introduction

 Pediatric traumatic brain injury (TBI) remains one of the leading causes of acquired disability and death, with the highest combined rates of TBI-related emergency room visits, hospitalizations, and deaths occurring in the youngest children [1]. Monitoring intracranial pressure (ICP) and cerebral perfusion pressure (CPP), repeat neurological assessments, CT scanning, and vital signs are considered the standard part of care following severe pediatric TBI. However, these assessments may not be sufficient to detect subtle and early secondary insults to the brain, such as ischemia, cerebral cytotoxic or vasogenic edema, and metabolic crisis. With the advent of newer methods of neuromonitoring, neuroimaging, and biomarker development, the concept of advanced neuromonitoring in neurocritical care has evolved in adults following severe TBI and is beginning to be explored in children. In this paper, we will discuss the role of advanced neuromonitoring in children following TBI.

## 2. Materials and Methods

We performed an extensive review of the medical literature regarding advanced neuromonitoring in children following TBI utilizing Pubmed. Search terms included “pediatric,” “child,” “neonate,” “brain,” “traumatic brain injury,” “multimodality,” “monitoring,” “brain oxygen,” “licox,” “jugular venous saturation,” “microdialysis,” “transcranial doppler,” “neuroimaging,” “diffusion weighted imaging,” “susceptibility weighted imaging,” “diffusion tensor imaging,” “ magnetic resonance spectroscopy,” “biospecimen,” “biomarker,” “common data elements,” “S100B,” “neuron specific enolase,” “myelin basic protein,” and “glial fibrillary acidic protein,” and the period of search was from 1987 to 2011. The authors are pediatric neurocritical care specialists and have extensive clinical experience caring for pediatric patients with TBI and research experience in clinical pediatric TBI and experimental animal models of pediatric TBI.

## 3. Results and Discussion

### 3.1. Neuromonitoring following Severe TBI in Children

#### 3.1.1. Brain Tissue Oxygen Monitoring

One of the primary management goals of the severely head injured child is to minimize secondary injury. One of the leading causes of secondary injury is cerebral ischemia and hypoxia [2]. Detecting cerebral ischemia with real-time continuous monitoring has proven to be elusive. Adherence to ICP- and CPP-driven management protocols does not guarantee the avoidance of cerebral ischemia or hypoxia [3, 4]. Cerebral blood (CBF) flow threshold for cerebral ischemia in the nontraumatized brain has been reported at 18 mL/100 g/min, and, when CBF falls below 10 mL/100 g/min, cerebral infarction occurs [5, 6]. In TBI, it appears the relationship between CBF and brain metabolism is much more complex. Injured tissues may have reduced metabolic needs due to cellular injury and/or mitochondrial dysfunction in which case lower CBF levels may be appropriate or tissues may be hypermetabolic and normal CBF levels may still result in secondary injury. The ability to measure adequacy of brain oxygen delivery and utilization can assist the clinician in managing the severe pediatric TBI patient to minimize the magnitude of secondary injury. The two most common methods utilized to assess brain oxygenation are brain tissue oxygen tension and jugular venous saturation monitoring, each with various strength and weaknesses.

#### 3.1.2. Brain Tissue Oxygen Tension Monitoring

 Brain tissue oxygen tension (PbtO_2_) monitoring has been utilized by clinicians to guide medical management of both adult and pediatric severely head injured patients. The most commonly used commercially available system is the Licox system (Licox, Integra Life Sciences, Plainsboro, NJ). A polarographic electrode catheter is inserted into the brain parenchyma of the region of interest using a specially designed bolt system. Oxygen diffuses across the catheter membrane and generates a voltage which is proportional to the amount of oxygen at the electrode site. The voltage-oxygen relationship is temperature dependent, and brain parenchymal temperature must be continuously monitored at the electrode site for accurate PbtO_2_ measurements. A combined oxygen electrode and temperature probe has been developed to reduce the number of ports required in the bolt system [7]. The probe surface area is approximately 1–15 mm^2^, so that information obtained represents only a very small region of brain parenchyma. This emphasizes the need for confirmation of probe location. Typically after insertion, an oxygen challenge is performed, inspired fraction of oxygen is temporarily increased to 100%, and a subsequent rise in PbtO_2_ should be observed [4, 8]. Computed tomography (CT) of the head can also be performed to assess probe location, but this need must be weighed with the risk of repeated radiation exposure to the pediatric patient [9].

 What the PbtO_2_ value physiologically represents is not completely clear and is still under investigation. In clinical studies, PbtO_2_ has been observed to vary with arterial oxygen tension as well as factors that affect CBF (such as arterial CO_2_ and cerebral perfusion pressure) [10–14]. Most likely, PbtO_2_ reflects the accumulation of oxygen in the brain parenchyma which is directly influenced by three factors: oxygen delivery, oxygen diffusion characteristics of the region of interest, and oxygen utilization by the brain tissue. Clinical studies have yet to establish normal and threshold values of PbtO_2_ for adults or children. Extrapolating from animal studies, normal PbtO_2_ values of 20–30 mm Hg are consistent with what has been observed in regions of uninjured brain clinically [15–17]. A threshold of 10 mm Hg has been associated with ischemic CBF levels, mitochondrial dysfunction, and derangements in cerebral metabolism as measured by microdialysis [6]. A persistent PbtO_2_ value of 0 mm Hg has been associated with brain death in several case series of both adults and children [18, 19]. PbtO_2_ values in the 10–20 mm Hg range may represent brain tissue at risk for secondary injury and may prompt the clinician to intervene.

 The association of PbtO_2_ < 10 mm Hg and poor outcome in TBI has been well documented in adults, but it remains unclear if PbtO_2_-directed management improves outcome [20–23]. Several adult studies have demonstrated improved outcomes following PbtO_2_-directed therapies compared to historical controls [24–26]. The first reported use of PbtO_2_-monitoring in children with TBI was a case series of six children reported in 2006 [27]. Since then there have been multiple reports of its use in pediatric TBI, but unfortunately it remains unclear if PbtO_2_-directed therapy improves outcome [3, 28, 29]. Some of the challenges in attempting to study the efficacy of PbtO_2_-monitoring include patient population and heterogeneity, the Hawthorne effect when using historical controls, and variations in interventions utilized in response to low PbtO_2_. Several reports on interventions to increase PbtO_2_ in children with TBI have recently been published [10, 30]. The effects of increasing arterial oxygen content and carrying capacity on PbtO_2_ have been evaluated in children with severe TBI [30]. Blood transfusions were observed to transiently increase PbtO_2_ in 17 children, but PbtO_2_ returned to baseline within 24 hrs. Normobaric hyperoxia challenges for 15 minutes in 28 children were observed to increase PbtO_2_, and a more robust response was observed to be associated with poorer outcome [10], but it remains unknown if normobaric hyperoxia should be instituted due to the concerns that an abundance of oxygen may result in increased free radical production and worsening secondary injury [31]. Due to the paucity of strong evidence for PbtO_2_ monitoring in pediatric TBI, the most recent consensus pediatric TBI guidelines could only make a level III recommendation to consider maintaining a PbtO_2_ ≥ 10 mm Hg if PbtO_2_ is used [32]. In summary, interpretation of PbtO_2_ monitoring in TBI remains complex, and effective and efficacious PbtO_2_-driven management protocols have yet to be established.

#### 3.1.3. Jugular Venous Saturation Monitoring

 Jugular venous saturation monitoring (SjvO_2_) is another method utilized to detect secondary insults following severe TBI. A catheter is inserted retrogradely to the jugular venous bulb. Catheter placement is confirmed with skull radiograph, with proper positioning demonstrating the catheter tip above the level of C1 [33]. Measurement can be obtained by analysis of blood samples or by utilizing a fiberoptic catheter to calculate jugular venous oxygen saturation. The incidence of complications associated with SjvO_2_ monitoring is low but can include bleeding, unintentional arterial puncture, thrombus formation, and infection.

 SjvO_2_ monitoring measures the balance between oxygen supply and demand. Factors affecting oxygen supply include oxygen carrying capacity, arterial oxygen content, oxygen-hemoglobin dissociation at the capillary level and CBF. Oxygen demand is primarily influenced by cerebral metabolic rate of oxygen (CMRO_2_). Increases in SjvO_2_ may reflect rises in CBF, reduced diffusion of oxygen into the tissues, or reduced CMRO_2_. Similarly, decreases in SjvO_2_ may be the result of cerebral hypoperfusion, decreased arterial oxygen content or carrying capacity, or increased CMRO_2_. The normal accepted range for SjvO_2_ in adults is 50–75%, but consensus thresholds for children have not been established. SjvO_2_ values below 50% in adult TBI patients have been associated with poor outcome [34], while SjvO_2_ values above 75% have also been observed to have a negative effect on outcome [35].

 The pediatric literature for the use of SjvO_2_ monitoring in children with TBI is quite limited. Pérez et al. reported their experience with 27 children with severe TBI who underwent SjvO_2_ monitoring [36]. They observed a significant association of two or more episodes of SjvO_2_ below 50% with poor outcome. However, SjvO_2_ values greater than 70% were not associated with poor outcome. There are several challenges and limitations to SjvO_2_ monitoring. Catheter location may vary with head positioning, catheter size may impede its use in younger children, and the development of thrombi can require frequent recalibration. In summary, the pediatric experience with SjvO_2_ monitoring is limited, normal ranges and SjvO_2_ thresholds for children have yet to be established and similar to PbtO_2_ monitoring, and the efficacy of SjvO_2_ driven management protocols has not been established.

#### 3.1.4. Cerebral Microdialysis

 Cerebral microdialysis is a neuromonitoring technique which has primarily been utilized as an invaluable research tool but holds potential to improve the care of the pediatric TBI patient. The technique involves the insertion of a semipermeable membrane tipped catheter into the brain parenchyma. The catheter is then infused with a dialysate at a low continuous rate (typically 1 microliter/min). Small molecules diffuse across the semipermeable membrane from the brain interstitial fluid into the dialysate. Samples can be collected at desired intervals and analyzed for various substances including markers of cell metabolism (lactate, pyruvate, glucose), neurotransmitters (glutamate), or markers of tissue damage (glycerol). The catheter membranes can have various molecular size cutoffs ranging from 20 to 100 kDa. Normal values for several small molecules have been established in adults [37]. Small adult studies have observed sustained elevations in the lactate/pyruvate ratios in pericontusional brain tissue [38]. Elevated brain tissue glycerol levels have also been observed to be associated with poor outcome following severe TBI in adults [39, 40]. A consensus published on microdialysis in adults recommended the use of lactate/pyruvate ratios, glucose, glycerol, and glutamate as markers for the detection of ischemia following TBI [41].

The first use of microdialysis in children with TBI was a case series of nine children in 2002 and focused on the levels of neurotransmitter amino acids (glutamate) as well as nonneurotransmitter amino acids (threonine, tryptophan, lysine, and tyrosine) [42]. Although a small sample size, the authors reported differences in the levels of excitatory neurotransmitters compared to previous reports in adults. This observation highlights the need to explore whether the changes in brain development (such as ongoing myelination) may result in variations in normal ranges and critical thresholds of microdialysis in pediatric TBI compared to adults. In summary, cerebral microdialysis is an exciting research tool which has not yet demonstrated proven benefit in the management of children with severe TBI.

#### 3.1.5. Transcranial Doppler Ultrasonography

Transcranial doppler ultrasonography (TCD) is a bedside test utilized to measure cerebral blood flow velocity typically of the middle cerebral artery. Accuracy of the method can be user dependent, and interobserver reliability can be variable. Cerebral blood flow velocities vary with age, and these changes correlate well with age-dependent changes in CBF [43]. TCD has been utilized as a noninvasive method to estimate CPP and ICP. Utilizing a pulsatility index (PI) under the assumption of stable blood pressure and arterial CO_2_ content, strong correlations have been observed between PI and elevations in ICP [44–47]. While pediatric TBI experience with PI is not as robust, several recent studies of 34 children with severe TBI revealed a poor correlation of between PI and ICP [48, 49]. TCD can also be used to diagnose cerebral vasopasm which appears to be more common in adult TBI compared to children [49–51].

More recently, TCD has been utilized to evaluate the presence or absence of pressure autoregulation (AR). Cerebral pressure autoregulation is the physiologic response in which the diameter of cerebral blood vessels varies with changes in systemic blood pressure to ensure a constant CBF. As mean arterial pressure (MAP) rises, cerebral blood vessels (typically the arterioles) constrict, and, as mean arterial pressure decreases, cerebral blood vessels dilate. This pressure reactivity is thought to remain intact over a range of MAP from 50 to 150 mm Hg in the adult uninjured brain [52]. An autoregulation index (ARI) has been developed to measure if the autoregulation response is intact by the following equation: ARI = %ΔeCVR/%ΔMAP, where the estimated cerebrovascular resistance (eCVR) is calculated by the ratio of MAP to cerebral blood flow velocity measured by TCD. An ARI value ≥ 0.4 is typically considered intact autoregulation. Autoregulation has been observed to be impaired in up to two-thirds of adults and 40% of children with severe TBI [53–56]. Impairment in autoregulation has been observed to be associated with poor outcome in adult and pediatric TBI patients, but it remains unclear if impaired autoregulation is an independent risk factor for poor outcome [57–59].

### 3.2. Neuroimaging following TBI in Children

#### 3.2.1. Diffusion Weighted Imaging

 While CT scanning may still be the fastest way to evaluate for acute life-threatening intracranial emergencies following pediatric TBI, there has been a tremendous advancement in different MRI techniques to better characterize specific details of the injured brain. Importantly, these different MRI modes detect aspects of TBI that may not be detected by CT or conventional MRI. One of these MRI techniques is diffusion weighted imaging (DWI). Images on DWI are sensitive to differences in the diffusion rate of water molecules and can detect vasogenic and cytotoxic edema. Increased diffusion is thought to occur with vasogenic edema due to increased water in the extracellular space where there is increased mobility, while restricted diffusion due to decreased water movement in the intracellular space is thought to be due to cytotoxic edema [60].

 DWI also can quantify water mobility by determining apparent diffusion coefficient (ADC) values, a measure of random water motion limited by cellular composition. ADC measurements are useful in detecting diffuse axonal injury (DAI) in pediatric TBI patients. Before DWI, it was difficult to detect axonal ischemia or damage in the pediatric brain due to the relatively high water content of the developing white matter tracts. DWI allows improved differentiation between the uninjured immature brain and injured area because the immature white matter tracts have less water restriction than mature brain or hyperintense ADC values compared to the adult brain, while injured brain regions usually have increased water restriction or hypointense ADC values relative to the uninjured immature brain [61].

 While there are numerous studies of DWI and ADC for lesion detection and outcome following TBI in adults, less is known about DWI and ADC in pediatric TBI. In one recent study, ADC values (obtained within 7 days of injury) in the peripheral white matter tracts were significantly reduced in children with severe TBI that had poor outcomes compared to children with severe TBI that had good outcomes at 6–12 months after trauma [62]. Furthermore, acute ADC values in the peripheral white and gray matter regions following pediatric TBI were inversely correlated with long-term (1–4 years) neurocognitive outcomes [63]. DWI has also been shown to allow earlier detection of acute cerebral ischemia than conventional CT or MRI as processes associated with ischemic injury result in greater diffusion restriction [61, 64]. This may be especially important in cases of suspected abusive pediatric TBI or nonaccidental trauma (NAT). Several studies have revealed that DWI and ADC detected lesions earlier and more extensive brain injury, especially ischemic lesions and lesions in the white matter tracts, than conventional MRI or demonstrated lesions when conventional MRI appeared normal in children with suspected NAT [61, 65–71].

#### 3.2.2. Susceptibility Weighted Imaging

 In pediatric TBI, hemorrhagic shearing lesions associated with DAI are a very common pathologic entity [72]. Unlike the large hemorrhages seen in contusions, epidural and subdural hematomas, conventional CT or MRI will usually not pick up these small shearing hemorrhagic lesions. Susceptibility weighted imaging (SWI) is a form of MRI that utilizes the paramagnetic properties of blood products (intravascular and extravascular deoxyhemoglobin, methemoglobin, and hemosiderin) based on their magnetic susceptibility effects in order to increase the visibility of microscopic hemorrhages [73, 74]. SWI has been shown to be superior in detecting hemorrhagic DAI after TBI in children compared to conventional MRI [75, 76]. In another study in children with TBI and DAI, the patients with lower initial Glasgow Coma Scale (GCS) or prolonged coma had a significantly increased average number and volume of hemorrhagic DAI lesions [77]. Furthermore, children who had worse outcome at 6–12 months after injury had significantly increased extent of hemorrhagic DAI lesions [77]. Recently, the increased extent of hemorrhagic DAI was associated with worse intelligence quotient (IQ) and neuropsychologic functioning in children and adolescents at 1–4 years after trauma [78].

 SWI may also prove valuable in the assessment of children with NAT. In a recent study, the presence of intraparenchymal brain microhemorrhages in infants with NAT using SWI was associated with worse initial GCS and poor outcome at 6 or more months than children without microhemorrhages [79]. Furthermore, the presence of microhemorrhages on SWI combined with evidence of ischemia on DWI was the most predictive of poor outcome while other radiological findings (extra-axial hemorrhages, skull or skeletal fractures) were not [79].

#### 3.2.3. Diffusion Tensor Imaging

 Another novel modality in MRI involves the use of diffusion tensor imaging (DTI), which is a more complex form of DWI. DTI takes advantage of the directionality of water diffusion in the human brain and allows analysis of the white matter tracts [80, 81]. Water diffusion is considered isotropic when motion is free and equal in all directions. In the normal brain tissue, there are physical boundaries that restrict water diffusion in the white matter tracts, with greater water mobility parallel to the axons and restriction of mobility perpendicular to the axons. This diffusion restriction is termed fractional anisotropy (FA) or the ratio of anisotropy to isotropy [82]; FA ranges from 0 to 1, where values closer to 0 represent isotropy or increased diffusion, for example, as a result of injury [83]. In contrast, values closer to 1 represent water diffusion more parallel to the white matter tracts in normal brain tissue. Water diffusion by isotropy is also measured by ADC [84, 85]. Although the association between FA and ADC in the white matter tracts are very complex and incompletely understood, in general FA is inversely related to ADC. Typically, higher FA and lower ADC values are associated with intact white matter tracts.

 Pediatric DTI studies done within weeks to years after TBI generally reveal reduced FA. In one study, FA was reduced in the corpus callosum (CC) in TBI patients compared to control patients; FA values were also lower with more severe injuries (GCS < 10 and positive MRI findings) than those with milder injuries (GCS > 10 and normal MRI findings) [86]. Another study revealed reduced FA and increased ADC in the white matter tracts of the TBI group compared to controls [87] and correlated with cognitive and global outcome [88]. Several studies revealed that DTI studies performed at a mean of 3 years after trauma revealed lower FA in the isthmus, genu, body and splenium of the CC; those that had higher FA values had better cognitive function and global outcome [89, 90]. Other studies revealed reduced FA following trauma in various white matter tracts [91–93] and associated with working memory and executive deficits [92, 93]. A different study revealed that the initial injury severity, as assessed by GCS, correlated with changes in the FA in the white matter tracts even at a minimum of 1 year after TBI [94]. In contrast, DTI studies obtained within 1 week after mild pediatric TBI had increased FA and decreased ADC suggestive of cytotoxic edema, compared to controls [95–97]. These DTI abnormalities were associated with more emotional distress, postconcussive symptoms and 30-minute delayed recall [95–97].

#### 3.2.4. Magnetic Resonance Spectroscopy

 Another neuroimaging technique that allows noninvasive analysis of neurochemicals and their metabolites in the brain involves magnetic resonance spectroscopy (MRS). While MRI uses signals from the proton nuclei of water to reconstruct anatomical images, MRS uses the protons located on neurochemicals within brain tissues. Several key brain metabolites measured by MRS include *N*-acetylaspartate (NAA), an amino acid synthesized in the mitochondria, that is a neuronal and axonal marker that decreases with neuronal dysfunction or loss [98]. Total creatine (Cr) composed of phosphocreatine and its precursor Cr are markers of intact brain energy metabolism. Total choline (Cho) which predominantly consists of phosphoryl and glycerophosphoryl Cho is a marker for membrane repair or synthesis, demyelination, or inflammation. Lactate is a result of anaerobic glycolysis and/or may be a response to release of glutamate [99]. Glutamate and glutamine are excitatory amino acid neurotransmitters that are released after TBI and may play a major role in neuronal death [100]. Myoinositol is an organic osmolyte in astrocytes and increases with glial proliferation [101].

 Several MRS studies have revealed decreases in NAA ratios suggesting neuronal dysfunction or loss and increased Cho/Cr suggesting DAI not only in areas of visible injury but also in “normal-appearing” brain after pediatric TBI [102–107]. A decrease in NAA in a visibly injured brain is probably caused by the primary impact, while a decrease in NAA in a “normal-appearing” brain may suggest DAI and subsequent Wallerian degeneration [101]. Studies have also revealed increased lactate, myoinositol, or glutamate/glutamine levels following pediatric TBI [105, 108, 109]. Some of these neurometabolite alterations have been found to be predictive of outcome. Reduced NAA ratios, increased Cho/Cr or lactate or myoinositol were associated with poor outcomes 6–12 months after injury [105, 108]. Interestingly, increased glutamate/glutamine levels had no correlation with good or bad outcome 6–12 months following pediatric TBI [109]; in part, this may have been due to the fact that most children with poor outcome were evaluated later than those with good outcome, potentially beyond the time frame for peak elevation of these excitatory amino acid neurotransmitters after injury [109].

 Additional studies demonstrated that early MRS predicted long-term (1–4 years) outcome after pediatric TBI. One study demonstrated that regional measures of NAA ratios obtained 1-2 weeks after TBI in children were associated with 40% variance in cognitive function obtained 1–4 years after injury [110]. Furthermore, regional NAA ratios were correlated with attention and executive function [110]. Another study found a correlation between right frontal white matter choline/creatine ratio and reaction time [102]. Another study demonstrated that left frontal white matter choline and creatine correlated with parental report of internalizing behavioral problems and medial frontal gray matter creatine and choline correlated with parental report of social competence [103].

While these various advanced MRI modalities can enhance the early evaluation of children following pediatric TBI, the main disadvantages are the need for transport of a critically ill patient to the scanner, the length of time it takes, the findings are representative of only a “fixed” moment in time, and the potential problem of MRI compatibility with certain neuromonitoring devices.

### 3.3. Biospecimen, Biomarker, and Common Data Elements

#### 3.3.1. Biospecimen and Biomarker

The FDA defines a biomarker as, “a characteristic that is objectively measured and evaluated as an indicator of normal biologic processes, pathogenic processes, or pharmacologic responses to a therapeutic intervention.” Human biospecimens can come from likely repositories: tissue, cerebral microdialysate, cerebral spinal fluid (CSF), urine, and blood. These biospecimens represent a critical resource of molecular data (genes, proteins, lipids, metabolites, etc.) and form the emerging field of proteomic and metabolomic analyses in pediatric TBI translational medicine. The study of specific biomarkers for proteomic and metabolomics (including the study of lipids) analyses will allow investigators the ability to identify and quantify injury, pinpoint cellular location, decipher molecular mechanisms, and ultimately develop targeted therapeutic interventions. We will not discuss the study of genomic biomarkers in pediatric TBI due to global ethical questions that still need to be further investigated, but genomic analyses in pediatrics deserve hypothesis-driven clinical research and dedicated collaboration that is starting to emerge in adult TBI [111]. In pediatrics, blood is the most common biospecimen used to study specific biomarkers in pediatric TBI due to its relative availability and ease of collection; however, CSF, urine, and microdialysate have all been reported. Some of the most well-studied biomarkers in pediatric TBI are discussed below.

#### 3.3.2. S100 Calcium Binding Protein B (S100B)

S100B is the most extensively studied biomarker in pediatric TBI, and while the mechanism of release is still unknown, it is likely that S100B is released by damaged glial cells and may also be actively secreted by activated glial cells following brain injury [112]. Initial studies examining reference ranges for S100B in children have shown variability with age and sex, confounding its use in children [113, 114]. However, in one pediatric TBI study, peak levels of S100B correlated with 6-month Glasgow Outcomes Scale (GOS) Extended Pediatric Score with a negative predictive value of 97% combined with a positive predictive value of 75% [115]. Additional studies have also shown some correlation with S100B and predictors of outcome after accidental and inflicted pediatric TBI; however, S100B continues to have limitations that hinder its acceptance as an isolated biomarker for prognostication pediatric TBI: it is found, outside the CNS, there is variability in the values depending on the timing of collection and comorbidities including cardiac arrest [113, 116–120]. As with other biomarkers prognostication of pediatric TBI outcomes may be improved with a panel of biomarkers, or combining with other predictors such as neuroimaging [121].

#### 3.3.3. Neuron-Specific Enolase and Myelin Basic Protein

Neuron-specific enolase (NSE) is released from neurons passively after cellular destruction [122]. NSE can also be detected in neuroendocrine cells and certain tumor cells, as well red blood cells and platelets. Clinical studies investigating levels of NSE as a biomarker after TBI have found that NSE is neither sensitive nor specific for injury severity following TBI [123]. While NSE has not been proven useful at this time as a biomarker for prognostication, it has been valuable to inform molecular mechanisms of injury following brain injury and help direct translational studies to investigate neuroprotective targets for secondary injury. NSE biomarker levels have similar profiles in pediatric inflicted TBI and pediatric hypoxic-ischemic brain injury, but not in pediatric noninflicted TBI, with delayed increase in serum levels suggesting prolonged neuronal death likely due to apoptotic pathways, and the important role that hypoxic-ischemic injury may play in child abuse and cardiopulmonary arrest [113, 124]. Damage to the axon may be measured by myelin basic protein (MBP), a protein that is found throughout the white matter tracts of the CNS. Similar to NSE, delayed increases in MBP were also found after pediatric inflicted TBI and after pediatric hypoxic-ischemic brain injury suggesting prolonged axonal damage.

#### 3.3.4. Glial Fibrillary Acidic Protein

Glial fibrillary acidic protein (GFAP) is an essential cytoskeletal protein of astrocytes. GFAP does not seem to be found outside the central nervous system, and, unlike S100B, when GFAP is found in the CSF or serum, it is likely to be due to cellular damage and not secretion [125]. In one study, GFAP levels in the serum and CSF were elevated in children with severe TBI. In addition, serum GFAP levels on day 1 postinjury correlated with functional outcome at 6 months and those patients treated with therapeutic hypothermia for TBI did not have significantly different levels as compared to those treated with normothermia [126].

Examples of commonly used biomarker blood tests in clinical medicine include troponin and brain natriuretic peptide (BNP) in cardiac disease. Unfortunately, the clinical study of biomarkers for TBI in children are limited and even the most frequently studied biomarkers (S100 calcium binding protein B, neuron-specific enolase, myelin binding protein) are not currently standard measurement for pediatric TBI. While all of these biomarkers have shown some correlation between concentrations and outcomes, they still lack the statistical power to be clinically useful. Cerebral proteomic and metabolomic analyses are an emerging field and, with advancing biotechnology, new biomarkers are being developed and researchers are using advanced statistical analysis, such as finite element modeling and combining two or more biomarkers with and without collaborating data, such as neurologic imaging to correlate with outcomes. The hope is that these advanced biomarkers in the future will help assess the degree of TBI, tailor therapy, guide rehabilitation, and aid caregivers, and families in difficult decisions.

#### 3.3.5. Common Data Elements (CDEs) for Biospecimens

Pediatric TBI is a global problem, with the World Health Organization predicting that by the year 2020 TBI will be the most common cause of death in children. Coordinated research and strength of evidence-based medicine require standardization of definitions and data elements to compare findings across studies and facilitate data sharing amongst institutions [127]. This has recently been extended to include recommendations for pediatric patients, including recommendations from an expert panel for best practice guidelines to standardize the quality and accessibility of biospecimens for pediatric TBI [128]. While there are no specific recommendations regarding specific biomarkers, this expert panel does outline guidelines that will allow for consistency across studies to develop a minimal set of measures and direction for additional biospecimen collection for hypothesis-driven studies. Core CDE (minimum) recommendations include the collection of an acute (<24 h postinjury) serum sample for proteomic and metabolomics analyses [128]. Supplemental CDE (greater depth and breadth of exploration) recommendations focus on serial collection of serum and CSF samples which will allow for study of injury progression and trajectory, including identification of evolving molecular derangement and eventual study of molecular-targeted interventions dependent on the temporal cascade of secondary injury. More controversial is an extended CDE (emerging study in pediatrics requiring validation) recommendation for cerebral microdialysis as a data element [128]. Cerebral microdialysis as described in previous sections has not been universally adopted in pediatric TBI; however, microdialysis is extensively used in adult patients as a measure of cerebral metabolism and cerebral bioenergetic dysfunction. Further recommendations focus on the specifics of biospecimen collection, processing, documentation, and storage. In the emerging field of cerebral biomarkers of injury, a common vernacular and scientifically based analyses will allow clinicians to pool resources and standardize the approach to study and intervene for our most at risk patients, pediatric severe TBI.

## 4. Conclusion

 Neuromonitoring technology is at an “infant” stage in pediatric TBI. While further studies are clearly needed to determine whether these modalities and advancement in technology with noninvasive monitors will allow early and reliable recognition of reversible secondary brain insults, the ultimate question is whether these new modalities will aid in treatment strategies that will positively affect outcome.
